# Using Google Trends Data to Track Healthcare Use for Hand Osteoarthritis

**DOI:** 10.7759/cureus.13786

**Published:** 2021-03-09

**Authors:** Samuel A Cohen, Thompson Zhuang, Michelle Xiao, John B Michaud, Lauren Shapiro, Robin N Kamal

**Affiliations:** 1 Orthopaedic Surgery, Stanford University School of Medicine, Stanford, USA; 2 Orthopaedic Surgery, Duke University School of Medicine, Durham, USA

**Keywords:** google trends, hand osteoarthritis, public interest

## Abstract

Background

Google Trends (GT) is a free tool that provides analysis of search traffic for specified terms entered into the Google search engine. In this study, we evaluate the association between public interest in hand osteoarthritis (OA) as determined by GT search volumes and healthcare usage related to hand OA.

Methodology

We compiled GT data from 2010 to 2017 for the following group of hand OA-related search terms: “hand osteoarthritis,” “hand arthritis,” “hand swelling,” “hand stiffness,” and “chronic hand pain.” Claims associated with hand OA codes were obtained from an administrative database (14.8 million patients) using International Classification of Diseases codes from 2010 to 2017. We performed trend analysis using univariate linear regression of GT data and hand OA claims. A month-by-month analysis of variation from yearly GT means was conducted for hand OA-related search terms.

Results

There was increased public interest in hand OA-related search terms from January 2010 to December 2017. Univariate linear regression of GT data for hand OA-related search terms compared with hand OA claims demonstrated a significant positive correlation (p < 0.001, r = 0.707). Peak public interest in hand OA-related search terms was observed in July, May, and June.

Conclusions

This study demonstrates the ability of GT to track healthcare use related to hand OA. Our data also add to the evidence for monthly variations in public interest related to hand OA. Clinics and surgery centers can employ GT data to anticipate resource utilization by hand OA patients.

## Introduction

Osteoarthritis (OA) currently affects more than 30 million adults in the United States [1]. Hand OA is characterized by pain and stiffness and most commonly affects the carpometacarpal, distal interphalangeal, or proximal interphalangeal joints. Approximately 50% of all women and 25% of all men will experience pain and stiffness associated with hand OA by the time they are 85 years old [2]. An aging U.S. and global population is expected to increase the incidence of hand OA diagnoses in the next several decades, with the average age of diagnosis approximately 61 years [3,4]. A greater incidence of hand OA will have considerable economic and resource utilization consequences as patients with hand OA tend to utilize more healthcare resources and accumulate greater costs than patients without OA due to the physical impairment associated with the condition [5-9]. As the number of patients affected by hand OA continues to rise, healthcare systems and hand surgeons will benefit from tools to anticipate healthcare usage related to hand OA in order to adjust resource allocation and best identify and address patients’ needs. The ability to anticipate healthcare usage can be especially valuable to orthopedic practices and surgery centers that have been forced to flex or furlough both staff and physicians as a result of the coronavirus disease 2019 pandemic [10,11].

The internet may be one route to track public interest related to hand OA. As the internet has become increasingly accessible in the United States and globally, people have turned to the world wide web to seek information about their health. Google, a search engine that handles more than 1.2 trillion searches per year (approximately 90% of all internet searches), is a popular destination for patients attempting to learn more about their medical conditions [12]. There are approximately 7 million health-related Google searches in the United States every day, which accounts to nearly 5,000 health searches per minute [13]. Google Trends (GT) is a free, open source tool that allows customizable analysis of search term volumes entered into the Google search engine [14]. Previously, the GT tool has been utilized in public health studies to track public interest in various healthcare topics ranging from interest in orthopedic elective procedures to interest in cancer screening [15,16]. In addition, GT has been shown to predict healthcare usage for a variety of surgical and non-surgical cosmetic procedures [17,18]. Although GT has been used to characterize trends in public interest for orthopedic conditions such as knee swelling and foot pain, to the best of our knowledge, there are no studies on the correlation between GT data and actual healthcare usage for hand conditions such as OA [19,20].

Characterization of the relationship between GT volumes and healthcare use and the ability of the GT tool to track actual healthcare usage related to hand OA will provide valuable information to hand surgeons and healthcare systems alike that hope to anticipate demand for hand OA treatment. This knowledge can inform subsequent staffing and resource allocation decisions that maximize efficiency, productivity, and improve patient outcomes. In this study, we aim to evaluate the relationship between public interest in hand OA as determined by GT search volumes and healthcare usage related to hand OA. We will also describe monthly patterns in public interest related to hand OA in the United States.

## Materials and methods

Google Trends analysis

GT analyses can be customized by search term, time period, and geographic location. After a search term is entered into GT and the appropriate temporal and geographic constraints specified, GT generates visuals and outputs that reflect the volume of a given search term relative to peak popularity within the defined time period, which is assigned a value of 100 [14]. The data are presented as relative search volume (RSV), which is computed as the percentage of searches of a term in a location during a specific period of time. An RSV value of 100 indicates the largest ratio between searches for a specific topic and the total amount of Google queries. A value of zero indicates that, at the specified time point, the proportion of queries for the search term was less than 1% of its peak RSV (RSV 100).

Search terms related to hand OA were chosen using the “related queries” feature of GT. The five most common search terms related to hand OA generated by the GT tool were the following: “hand osteoarthritis,” “hand arthritis,” “hand swelling,” “hand stiffness,” and “chronic hand pain.” Databases of search volumes over time were collected for each of the five aforementioned search terms. We used GT’s customizable filters to include searches within the United States from January 2010 to December 2017 for all selected terms. GT data were gathered on a monthly basis, with RSV reported each month from January 2010 to December 2017. After monthly RSV for each respective search term was noted, an overall monthly “Hand OA-Related Search Terms” RSV was calculated by averaging monthly RSV values of all five search terms studied. Monthly “Hand OA-Related Search Terms” RSV values were used in a linear regression with monthly hand OA claims.

Claims database analysis

In order to evaluate trends in healthcare usage related to hand OA, we queried a large, national administrative claims database (Pearl Diver Inc., Colorado Springs, CO, USA). This database consists of inpatient and outpatient records of 14.8 million patients on private, Medicare Advantage, or Medicaid managed care plans. We identified patients with hand OA using International Classification of Diseases, Ninth or Tenth (ICD9/10) Revision diagnostic codes (Table 1). Claims associated with hand OA were obtained from January 2010 to December 2017.

**Table 1:** ICD-9 and ICD-10 diagnosis codes. ICD, International Classification of Diseases

Hand OA-related ICD-9 and ICD-10 diagnosis codes	ICD-9-D-71504, ICD-9-D-71514, ICD-9-D-71524, ICD-9-D-71534, ICD-9-D-71594, ICD-10-D-M12541, ICD-10-D-M12542, ICD-10-D-M12549, ICD-10-D-M151, ICD-10-D-M152, ICD-10-D-M180, ICD-10-D-M1810, ICD-10-D-M1811, ICD-10-D-M1812, ICD-10-D-M1830, ICD-10-D-M1831, ICD-10-D-M1832, ICD-10-D-M184, ICD-10-D-M189, ICD-10-D-M19041, ICD-10-D-M19042, ICD-10-D-M19049, ICD-10-D-M19141, ICD-10-D-M19142, ICD-10-D-M19241, ICD-10-D-M19242

Statistical analysis

We performed an a priori sample size estimation based on Pearson’s correlation. To detect a 0.30 correlation between GT search volumes and hand OA claims at an α of 0.05 and 80% power, a total sample size of 96 data points was collected, with one data point representing average RSV value among our group of hand OA-related search terms for each month from January 2010 to December 2017. Univariate analysis was used to test the association of GT search volumes and hand OA claims, reporting Pearson’s correlation coefficient. We interpreted it using Evans’ classification: less than 0.20 is very weak, 0.20-0.38 is weak, 0.40-0.59 is moderate, 0.60 to 0.79 is strong, and 0.80 or greater is a very strong correlation [21]. As a secondary exploratory question, we conducted a month-by-month analysis of variation from annual GT means for the group of search terms analyzed in this study to more closely examine monthly trends in public interest in hand OA. Statistical significance was defined at p < 0.05.

## Results

Correlation of Google Trends search volumes and hand osteoarthritis claims in the United States

**Figure 1:**
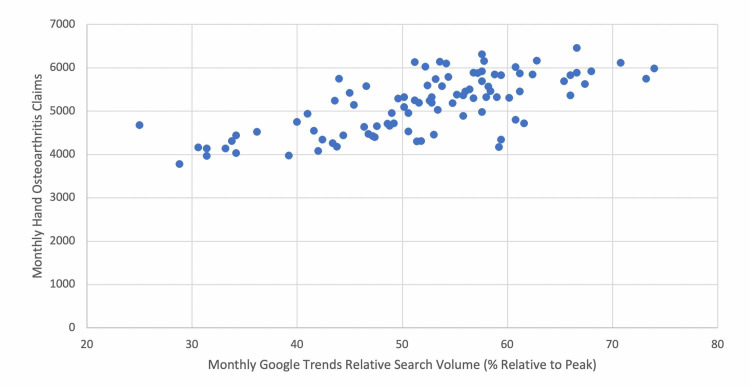
Scatterplot displaying monthly GT RSV for hand OA-related search terms and monthly hand OA claims, January 2010 to December 2017. GT, Google Trends; RSV, relative search volume; OA, osteoarthritis

Univariate linear regression of monthly GT data for hand OA-related search terms from 2010 to 2017 compared with monthly hand OA claims from an administrative claims database demonstrated a significant positive correlation (p < 0.001, r = 0.707). A scatterplot displaying monthly GT search volume and hand OA claims for all months from January 2010 to December 2017 is shown in Figure 1. In addition, Table 2 is a summary table displaying average annual GT search volume and annual hand OA claims from 2010 to 2017.

**Table 2:** Relationship of GT search volumes and hand OA claims, 2010-2017. GT, Google Trends; OA, osteoarthritis

Year	Mean GT search volume (% relative to peak)	Hand OA claims
2010	46.1	33,910
2011	50.8	37,923
2012	48.8	40,526
2013	54.9	44,518
2014	58.1	47,660
2015	61.8	48,621
2016	63.1	49,907
2017	72.4	51,231

Google Trends search volumes

**Figure 2:**
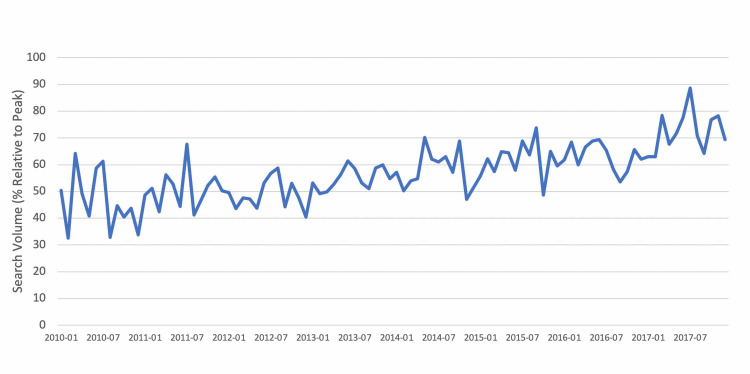
Mean GT RSV for hand OA-related search terms, January 2010 to December 2017. GT, Google Trends; RSV, relative search volume; OA, osteoarthritis

Overall, there was increase in public interest in hand OA-related search terms from January 2010 to December 2017. Mean monthly RSV values for hand OA-related search terms from January 2010 to December 2017 can be seen in Figure 2.

Monthly trends

**Figure 3:**
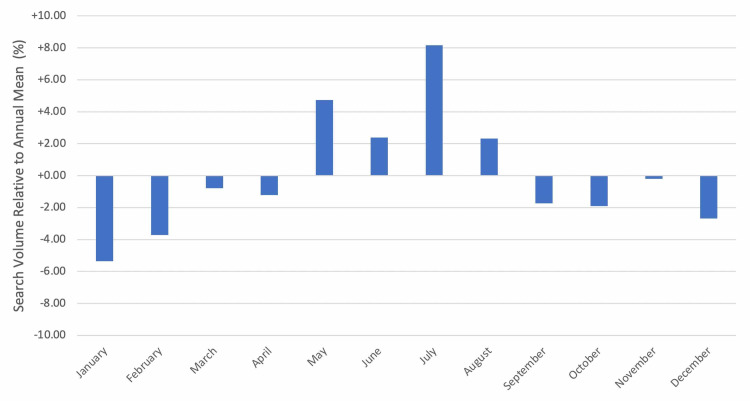
Monthly interest in hand OA-related search terms, 2010-2017. OA, osteoarthritis

When examining monthly trends in search interest for the group of search terms related to hand OA, peak interest was observed in the months of July (+8.16% relative to annual mean), May (+4.74%), and June (+2.39%). Public interest in hand OA-related search terms was the lowest in the months of January (-5.31%), February (-3.71%), and December (-2.69%) (Figure 3).

## Discussion

Our study demonstrates the ability of GT to track healthcare usage related to hand OA. We demonstrated significant positive correlation between GT traffic and hand OA claims for common search terms related to hand OA. This information may provide valuable insight to surgeons and health systems toward anticipating volume of hand OA procedures and for understanding which search terms are associated with claims, such as for marketing materials. Furthermore, we showed monthly variation in hand OA search volumes. GT data may be used by hand surgeons and health systems to track public interest in hand OA search terms, to predict trends in hand OA healthcare utilization, and to better identify and address patient needs.

Our findings add to the growing body of evidence suggesting that GT may be a powerful tool in helping physicians to anticipate and characterize healthcare usage related to medical conditions. Previously, the ability of GT to predict case volumes for both surgical and non-surgical cosmetic procedures has been documented [17,18]. Additionally, other GT studies have examined seasonal changes in public interest for a given search term such as “foot pain,” “ankle pain,” and “knee swelling” [19,20]. However, none of these studies correlated public interest as indicated by GT to actual healthcare usage for an orthopedic condition. The ability of the GT tool to track healthcare use for hand OA has wide implications as GT has the potential to provide real-time information about public interest in, and subsequent healthcare usage for, hand OA at the national, state, and even city level. Surgical centers treating patients with hand OA can monitor changes in public interest in hand OA to anticipate hand OA healthcare usage, which can inform future staffing and resource allocation decisions. Furthermore, the GT tool provides valuable information regarding the search terms that are most closely associated with hand OA claims, which can inform marketing materials. The GT tool is a convenient, free, and easily accessible tool that can be used to supplement more complex forecasting models in order to anticipate healthcare usage related to hand OA.

A positive correlation between GT search volumes for hand OA healthcare terms and healthcare usage suggests that hand OA patients are turning to the internet to learn more about their symptoms. Patients who are active on the internet have been shown to ask more questions during office visits than non-internet users, which can lengthen appointment times [22]. Hand surgeons hoping to validate patient questions and concerns without sacrificing clinic efficiency can take advantage of the fact that hand OA patients are using the internet to learn more about their condition and provide patients with handouts containing internet links to reputable sources of information that patients can engage with following their visit. These handouts can also help patients navigate the numerous hand OA resources on the internet to ensure decisions are made based on accurate information rather than information from web searches that is often inaccurate, incomplete, and overestimates the reading ability of the user [23-25]. Tech-savvy surgeons can even provide patients with reputable information about hand OA prior to office visits so patients can ask targeted questions that improve patient care and compliance without increasing appointment length [25]. In the future, investment in mobile applications and decision aids that utilize internet resources may be beneficial to patients and clinics alike.

Our results also indicate a monthly trend associated with Google searches related to hand OA, with greater public interest in hand OA in summer months and least public interest in winter months. It is possible that greater hand OA searches in the summer are a result of worsening hand OA symptoms due to seasonal activities that cause hand OA pain. Warmer summer temperatures may also contribute to worsening of hand OA symptoms. While the reason for greater public interest in hand OA during the summer is uncertain, GT provides actionable data that can be used by hand surgeons and surgery centers to anticipate future demand. In addition, GT data describing monthly hand OA search trends can be used in conjunction with the information provided by GT regarding which terms are most closely associated with hand OA claims to help hand surgeons devise marketing materials that optimize internet search traffic related to hand OA. Future studies may use the real-time data provided by GT to answer questions that still remain about the impact of weather conditions on hand OA symptoms, with many previous studies describing the relationship between weather and arthritis pain reporting conflicting results [26,27]. GT provides real-time, hourly information about Google searches related to hand OA symptoms that can be matched with real-time weather information to further clarify the relationship between weather conditions and hand OA symptoms.

There are several limitations to our study. First, although the Google search engine does capture a large majority of internet search traffic (approximately 90%), the GT tool cannot detect hand OA-related searches on alternate search engines [12]. Additionally, GT does not provide information about the demographics of the internet users whose data are reflected in this study, which makes it difficult to determine if Google users are representative of the U.S. population as a whole. Furthermore, the association between GT search volumes and hand OA claims volumes does not imply causation. Although the hand OA-related search terms demonstrated a significant, positive correlation to claims volumes, there are several other factors such as economic conditions, insurance status, and medical comorbidities that may affect whether a patient seeks treatment for hand OA symptoms [28-30]. Increased internet search traffic related to hand OA is only one of many factors that may contribute to an individual seeking treatment for hand OA. Finally, although the administrative claims database used to determine claims volumes contains information about 14.8 million patients on private, Medicare Advantage, or Medicaid managed care plans, these patients are not a nationally representative sample. Therefore, the trends described in our study should be interpreted with caution. Despite these limitations, our results demonstrate that the GT tool can be a powerful asset for hand surgeons and health systems that wish to anticipate healthcare use related to hand OA.

## Conclusions

In summary, our study demonstrates that GT search volumes associated with common search terms related to hand OA exhibit significant positive correlation with hand OA claims volumes. This information may provide valuable insight to hand surgeons and surgical centers that hope to anticipate volume of hand OA procedures for both staffing and resource allocation purposes. We also demonstrate which search terms related to hand OA are associated with claims, which can inform marketing materials. Furthermore, our results describe monthly trends in Google searches for terms related to hand OA, with increasing public interest in search terms related to hand OA in the summer and reduced public interest in the winter. The data provided by GT has the potential to provide actionable information that enable hand surgeons and surgery centers to better identify and address patient needs related to hand OA.
